# Prevalence of hyperprolific authors in sports medicine and musculoskeletal health and implications on research attention

**DOI:** 10.1371/journal.pone.0343827

**Published:** 2026-03-18

**Authors:** Serena Uppal, Haneef Khan, Michelle Helen Cruickshank, Michelle Ghert

**Affiliations:** 1 University of Galway, School of Medicine, Galway, Ireland; 2 University of South Carolina, School of Medicine, Columbia, South Carolina, United States of America; 3 McMaster University, Department of Surgery, Hamilton, Ontario, Canada; 4 University of Maryland, Department of Orthopaedics, Baltimore, Maryland, United States of America; Alma Mater Studiorum Universita di Bologna: Universita degli Studi di Bologna, ITALY

## Abstract

**Purpose:**

The phenomenon of hyperprolific authorship has raised concerns about research quality, academic integrity, and the sustainability of publication practices across scientific disciplines. Hyperprolific authors (HA) are defined as those publishing 72 or more papers annually, while almost hyperprolific authors (AHA) publish 61–72. This study aimed to identify and characterize extremely productive (EP) authors, defined as HA and AHA, in sports medicine and musculoskeletal health research and assess their scientific impact.

**Methods:**

We analyzed publications from the top 20 CiteScore-ranked journals in sports medicine and musculoskeletal health between 2020 and 2024 using the Scopus database. Authors were classified as HA or AHA based on annual publication volume. Metadata was extracted regarding publication counts, authorship positions, institutional affiliations, and geographic distribution. Citation impact and scholarly attention were evaluated using h-index and total citation counts.

**Results:**

Among 16,983 articles and 68,209 unique authors, 222 (0.45%) were classified as EP authors (125 HA, 97 AHA). Five authors maintained HA status across all five years, with the most prolific author publishing 1,174 papers and a peak annual output of 262. EP authors were concentrated in Europe (42.3%), Asia (28.4%), and the Americas (22.5%), especially in Germany, Japan, China, and the United States. Most EP authors were middle authors (median 59.8%–60.9%), with low first authorship (1.9%–2.1%) and higher last authorship rates (22.6%–27.0%). Despite concerns about volume, EP authors demonstrated substantial research attention, over their entire career, as measured by citation metrics: mean h-index 79.9 and mean total citations 35,654.

**Conclusions:**

Extremely productive authors comprise a small but influential subset of researchers. Their high output is not necessarily at the expense of research attention, but the concentration of productivity among a limited group raises important questions about authorship norms, research equity, and global representation.

## 1. Introduction

### 1.1. Background information and previous literature

Clinical research serves as the cornerstone for advancing the understanding of disease, fostering technological innovation, validating existing evidence, and expanding knowledge across a broad range of disciplines. As the volume of scientific publications continues to increase, growing concerns have been raised regarding the accelerating pace of article production and its potential impact on research. Many researchers face substantial pressure to produce new research due to the “publish or perish” model [1]. Metrics such as publication count and citation rates are commonly used to assess academic success and can significantly influence grant funding and career progression. While traditional citation counts and h-index have been widely adopted, newer, more nuanced metrics like the Relative Citation Ratio (RCR) have emerged to provide field- and time-normalized assessments of article influence, offering a more robust measure of scientific impact [2,3]. The RCR, developed by the National Institutes of Health, normalizes citations by comparing an article’s citation rate to an expected rate derived from its co-citation network, thereby accounting for field-specific citation behaviors.

Within this landscape, a growing focus has been placed on the phenomenon of hyperprolific authorship. Authors who consistently publish at exceptionally high rates have been classified as hyperprolific authors (HA), almost hyperprolific authors (AHA), or more broadly as extremely productive authors (EP), which encompasses both categories [4]. HA are commonly defined as those who publish at least one paper every five days, amounting to a minimum of 72 publications per year [5]. AHA fall slightly below this threshold, publishing between 61 and 72 papers annually, equating to roughly one article every six days [6].

The emergence of such authorship patterns has prompted critical discourse surrounding the integrity and scientific value of research outputs generated at such high frequencies. Hyperprolific authorship has prompted important questions regarding research practices, authorship ethics, and scholarly standards. Specifically, there is concern that such high rates of publication may reflect compromised research output, including superficial analyses, excessive fragmentation/redundancy of data, or honorary authorship practices. Alternatively, it is plausible that advances in research infrastructure, digital collaboration tools, and increasingly large multidisciplinary teams have enabled some authors to maintain high productivity without sacrificing scientific rigor. Distinguishing between these scenarios is crucial to understanding the true implications of hyperprolific publishing within contemporary research environments.

Systematic analyses aimed at identifying hyperprolific authors across various disciplines, followed by critical appraisal of their research impact, may provide valuable insights into these dynamics. If authors consistently produce high volumes of low-impact work, their designation as innovators or leaders within their respective fields warrants careful scrutiny. Recent investigations have begun to explore these issues in specific disciplines. For example, a study in intensive care medicine reported that the majority of hyperprolific authors in that field produced highly cited work and frequently published in leading journals. This could suggest that high productivity does not necessarily equate to low quality [7], however using citation counts to determine quality may not always be reliable [8]. Nonetheless, the authors of that study acknowledged the need for further research in other specialties to determine whether similar patterns hold elsewhere.

Recent analyses of hyperprolific authorship across 14 universities worldwide suggest that this pattern of publishing has become increasingly common. Between 2019 and 2023, publication rates rose by approximately 33% in the primary study group and by 20% in the control group [9]. The authors of that study reported not only a rise in the number of hyperprolific researchers but also a decline in first authorship and a corresponding inflation in total authorship counts. These outcomes align with broader publishing trends, as serving as first author often requires substantial time and effort, which can limit the overall number of papers an individual produces. When such responsibilities are reduced, the number of publications linked to a single author may rise, contributing to authorship inflation. The same study also observed a notable increase in collaboration among researchers and institutions at the international level [9]. Although this pattern may appear beneficial, the authors cautioned that much of the observed collaboration relied heavily on partnerships with researchers in other countries, often as a means to enhance publication output and visibility. A separate investigation focusing on the field of computer science reached similar conclusions. It found that hyperprolific authors tend to collaborate more frequently with others who share similar publication patterns, a dynamic that may help explain their accelerated publishing rates [10].

The present study aims to identify and characterize hyperprolific authors within the field of Sports Medicine and Musculoskeletal Health. By examining publication patterns and related metrics, this study seeks to assess whether hyperprolific authorship is prevalent in this subject area and to explore its potential impact on research within the discipline.

The phenomenon of HA and EP authors has been increasingly studied in recent years, largely due to the rapid growth of such authors across scientific disciplines. One landmark study analyzed publication trends across all scientific fields over a 23-year period, with particular focus on the dominance of hyperprolific authors within the field of physics [6]. This study further developed previous research which determined that the majority of HA are within the field of physics [5]. They assessed EP authors both including and excluding physics-related publications. Among non-physics disciplines, clinical medicine emerged as the leading contributor to the growing number of EP authors, with 1,661 HA and 2,543 AHA identified over the study period. In comparison, the physics group displayed significantly higher counts of EP authors, with most hyperprolific activity concentrated in this field.

The study also revealed temporal trends: while hyperprolific authorship in physics peaked between 2010 and 2012 and later declined, the number of EP authors in non-physics fields—including clinical medicine—showed steady growth throughout the entire period. By 2022, the prevalence of EP authors in clinical medicine approached levels comparable to those observed in physics. Geographically, the United States was the most represented country among EP authors in physics, whereas China dominated in non-physics fields.

Further focused investigation into clinical research productivity was conducted in a study of authors publishing in intensive care medicine between 2019 and 2023 [7]. This analysis identified 248 EP authors among 186,150 total authors across 42,860 articles. Among these EP authors, 131 were classified as HA and 117 as AHA. Most EP authors in this field were based in Europe—particularly Germany—and the majority were male, consistent with prior studies on author demographics.

Notably, authorship position analysis showed that EP authors rarely appeared in first-author positions, instead more frequently occupying middle or last authorship roles, reflecting patterns of seniority and supervisory responsibilities. Additionally, collaboration was common: approximately 92% of EP authors co-authored papers with at least one other EP author, suggesting the importance of established research networks in sustaining high productivity.

## 2. Materials and methods

### 2.1. Author identification

For this study, relevant authors were identified based on their publication activity in the top 20 orthopaedic journals indexed in Scopus between January 1, 2020 and December 31, 2024. Scopus is a comprehensive abstract and citation database of peer-reviewed literature. The target journals were selected according to their *CiteScore* rankings, which reflect the average number of citations per document over a four-year period.

The target journals included:

*British Journal of Sports Medicine*, *Sports Medicine*, *Journal of Sport and Health Science*, *Journal of Cachexia, Sarcopenia and Muscle*, *Journal of Orthopaedic Translation*, *Osteoarthritis and Cartilage*, *Journal of Bone and Mineral Research*, *Exercise and Sport Sciences Reviews*, *Bone and Joint Journal*, *American Journal of Sports Medicine*, *Arthroscopy: Journal of Arthroscopic and Related Surgery*, *Skeletal Muscle*, *Journal of Bone and Joint Surgery*, *Physical Education and Sport Pedagogy*, *Biology of Sport*, *Spine Journal*, *Knee Surgery, Sports Traumatology, Arthroscopy*, *Calcified Tissue International*, *Scandinavian Journal of Medicine and Science in Sports*, and *Annals of Physical and Rehabilitation Medicine*.

We retrieved all full-length articles published in these journals between 2020 and 2024 using the Scopus Search API with the following query:

“PUBYEAR > 2019 AND PUBYEAR < 2025 AND (DOCTYPE (ar) OR DOCTYPE (ip) OR DOCTYPE (re)) AND SOURCE-ID”.

Here, “SOURCE-ID” refers to the unique Scopus source identifier for each of the 20 selected journals. The document types representing full papers included “article,” “review,” and “article in press.” Other document types such as “editorial,” “note,” “letter,” “correction,” and “conference papers” were excluded from analysis.

From the retrieved publications, we extracted the complete list of contributing authors with valid Scopus Author IDs. This process yielded a comprehensive set of authors who had published at least one article in the selected orthopaedic journals during the study period.

To assess the research productivity of each identified author, we performed additional searches in Scopus to retrieve their full publication records, including articles published outside the orthopaedic field. This enabled calculation of the total number of publications across all subject areas for each author.

For each retrieved publication, we extracted detailed metadata including Scopus Author ID, publication title, keywords, publication year, journal title, number of co-authors, citation count, and institutional affiliation. All authors with valid Scopus Author IDs listed on these publications were identified and included for further analysis.

Authors were classified according to productivity thresholds previously reported in the literature. Specifically, hyperprolific authors (HA) were defined as those with an average publication rate of at least one article every five days, and almost hyperprolific authors (AHA) as those publishing at least one article every six days. Authors meeting these thresholds were further analyzed to assess their contributions to orthopaedic research, including evaluation against criteria for extreme productivity (EP) authorship which consists of both HA and AHA.

### 2.2. Data extraction

Annual publication counts were aggregated to examine trends in author productivity from 2020 to 2024. Key metrics, including total publication counts, annual publication distributions, authorship position, h-index, and author geographic profile were derived using the Scopus Search API and Scopus Author Retrieval API. The h-index and total citation counts retrieved from Scopus represent each author’s entire career metrics, not limited to the 2020–2024 study period. All data retrieval and processing from the Scopus and PubMed databases were conducted using custom Python scripts (Python 3.12, Python Software Foundation), employing the *requests* library. The code used in this study is publicly accessible and is available at github.com/HkKhan/EP-authorship-analysis.

### 2.3. Statistics

Descriptive statistics, including means, medians, standard deviations (SD), interquartile ranges (IQR), ranges, and percentiles, were calculated to summarize continuous variables such as total publication counts, annual publication rates, h-index, and total citation counts. Categorical data, including authorship positions and geographic distributions, were presented as frequencies and percentages. All statistical analyses were performed using custom Python scripts utilizing the *pandas*, *numpy,* and *seaborn* libraries for statistical computations and data presentation.

## 3. Results

Between 2020 and 2024, a total of 16,983 articles were published across the top 20 *CiteScore*-ranked journals in the fields of sports medicine and musculoskeletal health, with a median output of 849 articles per journal over this five-year period. From these publications, 68,209 unique authors with valid Scopus author identifiers were identified, representing the active author population contributing to orthopaedic research within this domain.

Among these authors, 222 individuals (0.45%) were classified as extremely productive (EP), with 125 designated as hyperprolific authors (HA) and 97 as almost hyperprolific authors (AHA) for at least one calendar year within the study period. The highest concentration of EP authors was observed in 2021, with 127 EP authors (73 HA and 54 AHA), followed by 2022 with 124 EP authors (71 HA and 53 AHA). In comparison, the years 2020, 2023, and 2024 recorded lower numbers of EP authors, with 85, 83, and 99 respectively.

Of the total EP cohort, 103 authors (46.4%) achieved EP status in only a single year during the observation period. This analysis was restricted to the 2020–2024 timeframe; we did not assess whether these authors had achieved EP status in years prior to 2020. The single-year EP designation indicates only that these authors met EP thresholds in one year within our study window, not necessarily that this represented their first or only year of extreme productivity across their entire careers. The prior years were not considered in order to maintain a consistent study period, allowing for fair comparison among authors, and to ensure that the results from this study are recent and relevant. However, the median duration of EP status was 2 years (range: 1–5 years). Notably, five authors consistently maintained HA status throughout all five years (EP Authors 1–5), demonstrating sustained high productivity. These most prolific individuals authored between 613 and 1,174 articles during the study window, with peak annual outputs ranging from 115 to 262 publications per year.

### 3.1. Hyperprolific and almost hyperprolific author analysis

**Fig 1:**
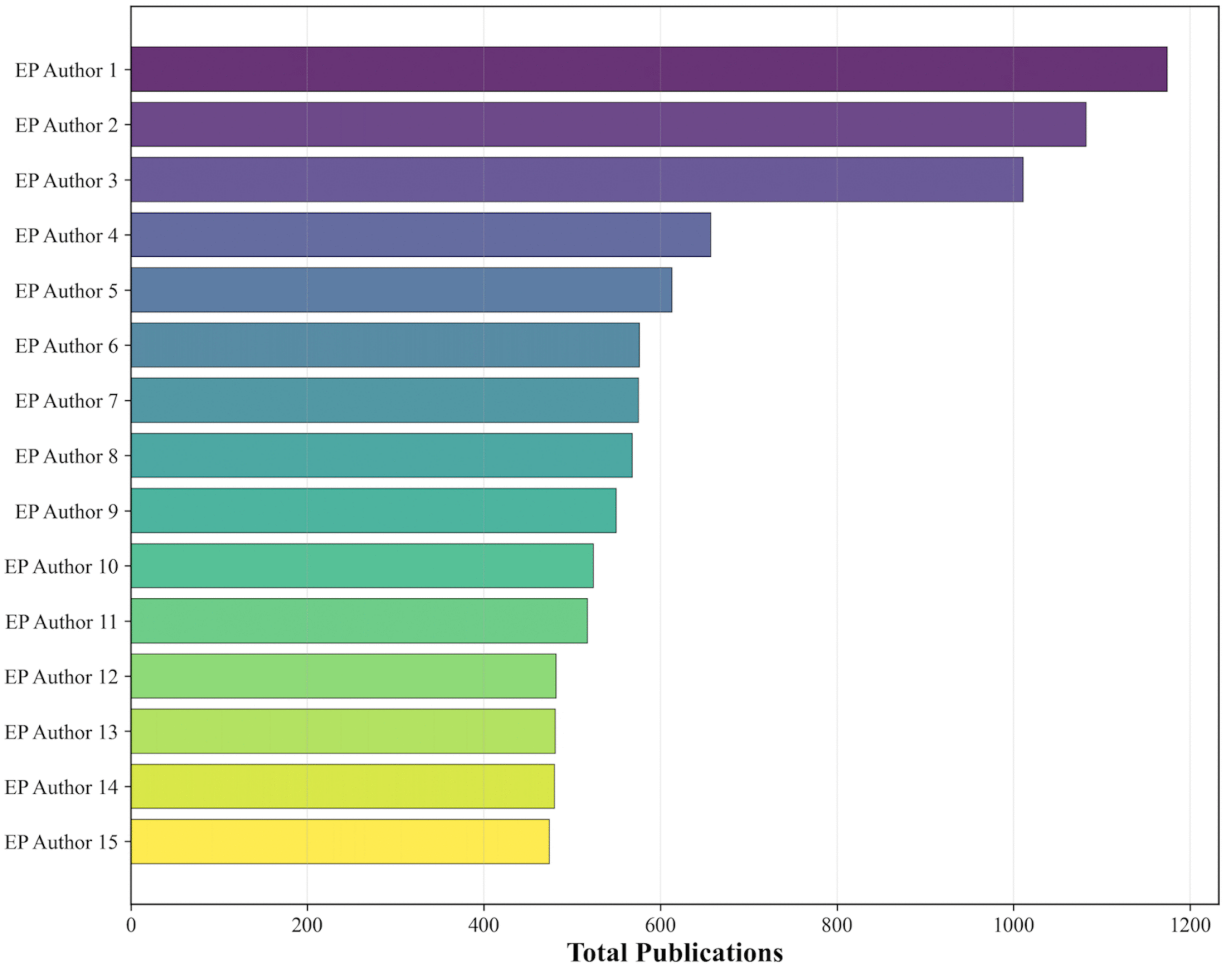
Productivity of the top 15 EP authors, including the total number of publications throughout the four-year time period. Overall among all EP authors, there was a median of 15 global publications per author. The mean number of publications per author was 26.5 (SD = 35.7), with a range from 1 to 1,174 publications. The publication count percentiles were: 25th percentile at 7 publications, 75th percentile at 32 publications, 90th percentile at 61 publications, 95th percentile at 89 publications, and 99th percentile at 171 publications.

Among the 125 HA authors, the most prolific was EP Author 1, with 1,174 total publications and a maximum annual output of 262 articles. Other leading HA authors included EP Author 2 (1,082 publications; peak annual output: 235), EP Author 3 (1,011; peak: 246), and EP Author 4 (657; peak: 158). The results of the top fifteen authors are outlined in Fig 1.

For the 97 AHA authors, the most productive was AHA Author 1, who produced 329 publications and maintained AHA status for four consecutive years. Other high-output AHA authors included AHA Author 2 (322 publications, AHA for 4 years), AHA Author 3 (316 publications, AHA for 3 years), and AHA Author 4 (314 publications, AHA for 3 years).

### 3.2. Geographic distribution

Geographically, EP authors were primarily located in Europe, Asia, and the Americas. Of the 222 EP authors, 94 (42.3%) were based in Europe, 63 (28.4%) in Asia, and 50 (22.5%) in the Americas. Minor contributions were noted from Oceania (6 authors; 2.7%) and Africa (3 authors; 1.4%). Within continents, the most represented nations were Germany (31 authors) in Europe, Japan and China (24 authors each) in Asia, and the United States (45 authors) in the Americas.

### 3.3. Authorship position analysis

Authorship position analysis revealed similar patterns between HA and AHA authors. Among HA authors, first authorship comprised a median of 2.1% of their publications (interquartile range [IQR]: 1.4%–2.7%), whereas last authorship accounted for a median of 27.0% (IQR: 15.0%–40.0%). The majority of their publications were in middle authorship positions, with a median of 59.8%.

**Fig 2:**
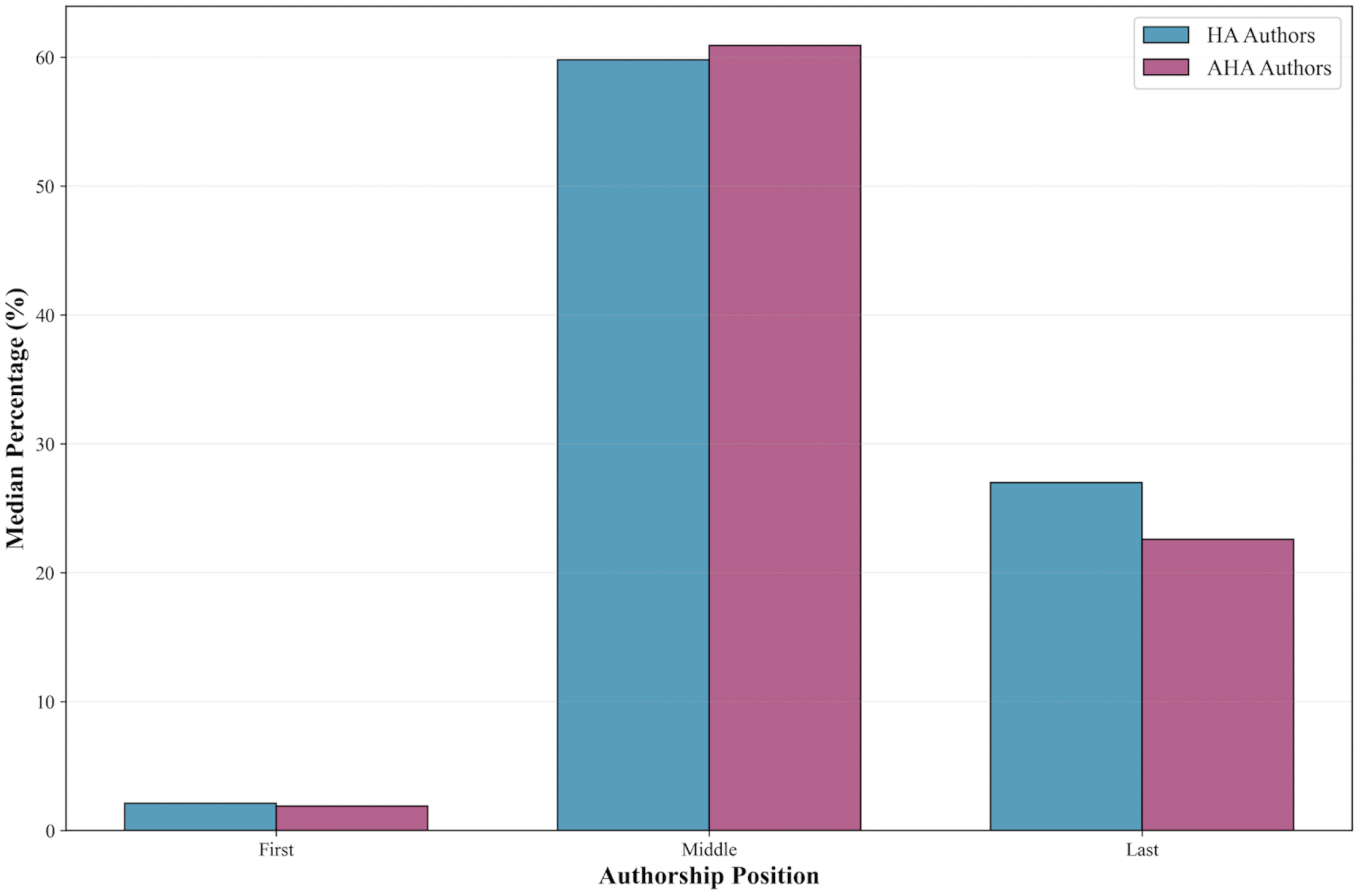
Comparison of authorship position between HA and AHA. Similar trends are observed for both groups.

Similarly, AHA authors exhibited comparable trends, with first authorship representing a median of 1.9% (IQR: 1.1%–2.8%) and last authorship comprising 22.6% (IQR: 14.3%–37.5%). Middle authorship positions dominated among AHA authors as well, representing a median of 60.9% of their works. Authorship position can be compared among HA and AHA as seen in Fig 2.

### 3.4. Research attention: Citation and H-Index analysis

Citation analyses demonstrated substantial differences between HA and AHA authors. HA authors achieved a mean h-index of 87 (median: 71.0; IQR: 46.0–118.0; SD: 54.9) and a mean total citation count of 43,567 (median: 20,934). In contrast, AHA authors had a mean h-index of 70.9 (median: 58.0; IQR: 42.0–97.0; SD: 47.6) and a mean total citation count of 25,457 (median: 11,592).

When considering the full EP cohort (n = 222), the group attained a mean h-index of 79.9 (median: 63.5; IQR: 43.0–109.8; SD: 52.5) and mean total citations of 35,654 (median: 15,160). It is important to note that the h-index has known limitations as a metric of research impact. While widely used, it does not distinguish between positive, negative, or neutral citations, may favor established researchers with longer careers, and does not capture the full breadth of scholarly contributions. Additionally, high h-index values reflect research attention rather than direct measures of methodological rigor or research quality.

### 3.5. Publication and productivity patterns

Publication distribution exhibited a markedly right-skewed pattern. Authors producing ≥200 publications represented only 0.6% of the total author population yet accounted for a disproportionately high share of total publications. The median number of publications per active author was consistent at four across all five years. The annual number of active authors ranged from 38,545 in 2020–42,362 in 2022.

Annual publication trends revealed minor year-to-year variations. The peak publication year was 2021, with 277,848 articles authored by 41,493 active authors, corresponding to a mean of 6.7 articles per active author. This was followed by 2022 (276,110 articles, 42,362 authors; mean: 6.5 per author). Publication volumes were relatively lower in 2020 (236,143 articles), 2023 (254,291), and 2024 (251,872), with mean publications per author ranging from 6.1 to 6.3.

The proportion of authors achieving EP status remained relatively stable, peaking at 0.26% in 2021 and reaching a low of 0.17% in 2023. Notably, 20 authors (16% of all HA authors) maintained HA status throughout the entire study period.

## 4. Discussion

### 4.1. Existence of extremely productive authors

This study demonstrates that EP, HA represent a small minority within the broader authorship landscape of sports medicine and musculoskeletal health research, comprising just 0.45% of the total author population. Although the comparative numbers of HA and AHA remained relatively balanced throughout the five-year study period, year-to-year fluctuations were observed. Notably, nearly half of the EP authors maintained this status for only a single year, suggesting variability in research output and potentially reflecting temporal shifts in research engagement, access to funding, or strategic prioritization of publications during specific years. This observation may also indicate that for some authors, prolific publishing is driven by temporary circumstances such as project-based funding, academic promotion cycles, or involvement in large-scale collaborative studies.

Conversely, a small subset of EP authors demonstrated sustained productivity, maintaining HA status across the entire five-year study window. These consistently prolific individuals represent an elite group characterized by remarkable and sustained research output. In contrast, AHA authors displayed greater variability in EP status duration, often maintaining high productivity for shorter periods relative to HA authors. This difference may reflect divergent academic roles, institutional expectations, or the degree of involvement in collaborative networks, where some authors may serve as transient contributors to large research groups while others maintain sustained leadership positions.

### 4.2. Global distribution of extremely productive authors

This analysis examines the scope of orthopaedic and sports medicine research, in English-language collections, among EP authors distributed across multiple continents. However, the majority of EP authors were affiliated with institutions in Europe and North America. These findings are consistent with previous bibliometric analyses, which have shown that Europe contributed nearly one-third of all publications in orthopaedics and sports medicine between 1996 and 2022 [11]. Our results further highlight Europe’s continued prominence as a leading hub for musculoskeletal research, with Germany emerging as the most represented country within Europe, while the United States accounted for the largest overall number of EP authors globally. Both countries have well-established research infrastructures and long histories of high publication output in orthopaedics [12], contributing to their dominant presence within this analysis.

These geographic findings must be interpreted with caution due to inherent biases in English-language scholarly indexing. Major citation databases including Scopus, Web of Science, PubMed, and Embase disproportionately index English-language journals, creating a systematic bias toward documenting research from English-speaking regions and the Global North [13]. Consequently, our analysis captures EP authorship patterns only within the English-language publishing ecosystem indexed by Scopus. We cannot determine whether EP authorship is predominantly a Global North phenomenon or whether similar patterns exist in other language-based research ecosystems that remain underrepresented in major English-language indexes. The absence of documented EP authors from certain regions may reflect indexing bias rather than absence of such authors. Future research should examine EP authorship patterns in non-English-language journals and regional databases to provide a truly global perspective on this phenomenon.

### 4.3. Authorship roles and publication strategies

Authorship position analysis provides valuable insight into the research profiles and collaborative practices of EP authors. The majority of EP authors occupied middle-author positions in their publications, with relatively low representation as first authors—a role typically associated with primary contributions to study design, data collection, and manuscript preparation. The low proportion of first authorship among EP authors suggests that many of these individuals may participate in numerous projects in a secondary or collaborative capacity rather than leading each study.

The predominance of middle authorship positions (approximately 60% for both HA and AHA) raises additional questions about the nature of EP authors’ contributions. While senior researchers may legitimately contribute to multiple concurrent projects in advisory or collaborative capacities, the relatively modest proportion of last authorship positions (22–27%) compared to middle positions suggests that supervisory roles alone may not fully explain hyperprolific output [14,15]. Middle authorship positions could reflect: (1) established researchers whose reputations lead to inclusion on manuscripts to enhance visibility, (2) genuine collaborative contributions across multiple research networks, or (3) practices that may warrant scrutiny regarding honorary or guest authorship [16,17]. The short five-year timeframe of our analysis, combined with the concentration of middle rather than last authorship, suggests that honorary authorship practices may contribute to some EP patterns and merit further investigation.

In contrast, in some cases the EP authors occupied last authorship positions, which is traditionally associated with senior supervisory roles or principal investigatorship. This pattern aligns with expectations, as senior researchers often oversee multiple concurrent projects, enabling them to contribute to many publications simultaneously.

Such dynamics are partially consistent with findings from prior studies in other disciplines, including intensive care medicine, where EP authors similarly predominated other authorship positions (not including first, second or last), with the next most common being last authorship [7]. The ‘other authorship positions’ can be presumed to be similar to middle authorship. These patterns suggest that sustained high productivity may be closely linked to supervisory or collaborative roles within large research networks [18,19].

### 4.4. Citation impact

Concerns are often raised regarding whether high publication volumes may compromise research attention. To address this, we evaluated the h-index as an indicator of citation frequency and scholarly attention. However, it is critical to note that citations reflect research attention rather than research quality. Citations can be positive, negative, neutral, or rhetorical in nature. Research quality can only be assessed through evaluation of methodological rigor, completeness of reporting, and adherence to statistical and scientific standards—aspects not measured by citation counts or the h-index.

The h-index, which balances both publication count and citation frequency, is widely regarded as a robust indicator of academic impact, comparing the number of published articles to the number of associated citations [20]. Across all EP authors, the mean h-index was 79.9, indicating that these authors had produced at least 79 publications each cited at least 79 times—an exceptionally high level of scholarly influence.

For reference, Hirsch originally suggested that an h-index above 60 might represent substantial scholarly attention over a career [21]. However, this threshold was proposed before the phenomenon of hyperprolific authorship was documented and does not account for authors publishing hundreds of papers annually. Furthermore, the h-index has faced criticism, including from its creator, regarding its limitations in capturing the full impact of an author’s corpus, particularly the contribution of papers beyond the h-core [22]. These findings suggest that EP authors in sports medicine and musculoskeletal health research do not merely achieve high publication counts, but also produce work that receives substantial attention as measured by citations. However, citation frequency reflects research attention rather than direct evidence of methodological quality [23,24]. High citation counts indicate that work is noticed and referenced, but do not necessarily validate methodological rigor, completeness of reporting, or freedom from bias. A comprehensive assessment of research quality would require systematic evaluation of study design, statistical methods, and adherence to reporting standards—analyses beyond the scope of the present study. The combination of high productivity and high citation impact underscores the significant contributions of these authors to the field.

### 4.5. Equity considerations and broader implications

Although this study did not include detailed demographic analysis, prior research has consistently demonstrated that EP authors are disproportionately male and predominantly affiliated with institutions in high-income countries [7]. These trends mirror broader patterns in academic medicine, where men remain overrepresented in senior and leadership roles, particularly within surgical and procedural specialties such as orthopaedics [25,26].

Such disparities raise important questions regarding equity in research careers, access to mentorship, and the distribution of institutional resources that enable sustained high productivity. However, the current study did not consider alternative research environments, therefore acknowledging these disparities will require further assessment of EP authorship in various areas. The analysis in other regions, outside of sports medicine including a range of new subject areas, is a necessary next step for the development of this field, allowing for comparison of publication numbers between research areas for the same author. Additionally, determining the dispersion of publication areas for each author can help shape future analyses. For example, the increased number of COVID-19 related papers during the pandemic written by authors with little experience in the field could contribute to inflated publication rates. Furthermore, the dominance of EP authors from high-income countries may reinforce existing global inequities in academic visibility, research funding, and influence [27,28]. Addressing these structural barriers is essential to promoting greater diversity and inclusion in orthopaedic research and ensuring that opportunities for scholarly achievement are equitably distributed.

### 4.6. Implications

The findings of this study have several important implications for orthopaedic research and academic publishing more broadly. Notably, this is the first study to systematically identify and characterize EP authors in the field of sports medicine and musculoskeletal health, establishing a foundation for future investigations within this specialty. While concerns have been raised that high publication volume may compromise research attention, our results suggest otherwise. EP authors demonstrated consistently high citation impact, as reflected by elevated h-index scores, indicating that prolific publishing can coexist with influential and well-cited research output in orthopaedics. However, the concentration of research output among a small group of authors raises questions regarding the distribution of research opportunities, authorship equity, and collaborative practices.

Importantly, EP authors comprise less than 0.5% of the total author population in sports medicine and musculoskeletal health research. This small proportion suggests that hyperprolific authorship is not a systemic or endemic issue within the field. The majority of researchers publish at conventional rates, and EP authors alone cannot substantially skew overall publication metrics. However, the concentration of publications among a small group does raise questions about resource allocation, particularly regarding article processing charges (APCs) in open-access journals [29,30]. A small number of authors generating hundreds of publications annually may disproportionately consume institutional and grant funding that could otherwise support broader research diversity. Additionally, while EP authorship patterns could theoretically be moderated through editorial oversight and rigorous peer review [31], journals must balance quality control with the practical and financial incentives of increased publication volume.

The predominance of EP authors in senior or supervisory authorship roles further highlights the hierarchical nature of research in this field, with implications for mentorship and access to leading authorship positions among early-career researchers [32,33]. Additionally, the geographic clustering of EP authors in high-income countries underscores persistent global disparities in research capacity and visibility, suggesting the need for policies that promote greater inclusion of underrepresented regions. Finally, these findings call for continued monitoring of hyperprolific publishing patterns to ensure that high productivity remains aligned with scientific integrity.

These findings should be considered within the broader context of ongoing reforms in research assessment practices. Initiatives such as the Declaration on Research Assessment (DORA), the Agreement on Reforming Research Assessment (ARRA), and the Coalition for Advancing Research Assessment (CoARA) have emphasized moving away from journal-based metrics and citation counts as primary indicators of research quality [34,35]. The concentration of publications among EP authors, while associated with high citation impact, underscores the need for more nuanced evaluation frameworks that consider diverse contributions to scholarship beyond raw publication volume and citation metrics.

### 4.7. Strengths and limitations

This study has several strengths and limitations that should be considered. To our knowledge, it is the first to systematically identify and characterize hyperprolific authors within the fields of sports medicine and musculoskeletal health, providing novel insights into authorship patterns, geographic distribution, and citation impact in these disciplines. By incorporating h-index analysis, the study also offers valuable perspectives on the relationship between publication quantity and research influence—addressing common concerns about the potential trade-off between productivity and impact. In addition, the use of Scopus author identifiers helped to minimize author misattribution and ensured consistency in author tracking [36].

However, several limitations warrant discussion. The analysis was limited to the top 20 CiteScore-ranked journals, which may have excluded productive authors who primarily publish in other reputable outlets. Compared to studies in other specialties, the smaller number of included journals reflects the lower publication volume in orthopaedic research, potentially limiting the identification of additional EP authors and reducing generalizability across subfields. All publication types were included without distinguishing between original research, reviews, or other article types, despite differences in scientific contribution. While Scopus provided a standardized data source, reliance on a single database may have excluded authors indexed elsewhere [36]. Furthermore, citation-based metrics such as the h-index may favor senior or established researchers and do not fully capture research novelty or societal impact [37]. Additionally, our analysis excluded editorials, notes, letters, corrections, and conference papers, focusing only on full-length articles and reviews. While this approach enabled examination of substantive research contributions, it prevented assessment of whether EP authors disproportionately utilize shorter communication formats to inflate publication counts. An analysis comparing EP authors’ output across document types could help distinguish genuine hyperprolific authorship (predominantly empirical papers) from artificial productivity (disproportionate reliance on commentaries, perspective pieces, and other rapid-publication formats). Future studies should examine the document type distribution among EP authors to better characterize their publication strategies. The use of H-index can also provoke some concern regarding the validity of the metric. The sheer number of citations may not always indicate the impact of the reference, as well as the contributions of individual authors [38]. In future studies, to attempt to combat this limitation, the excess index (e-index) can be calculated for each author [39]. This measure can provide insight on the strength of each paper beyond the scope of the h-index. Lastly, the study did not account for demographic variables such as gender, career stage, or institutional characteristics, limiting assessments of diversity and equity within the author population.

### 4.8. Future directions

Several avenues for future research emerge from this study. Given the relative stability of annual publication trends observed in this study, future research should examine the thematic content and methodological characteristics of EP authors’ output. Qualitative analysis of research topics, study designs, and methodological rigor—including assessment of adherence to reporting guidelines, statistical adequacy, and risk of bias [40,41]—would help distinguish between diverse high-quality contributions and potentially fragmented or methodologically limited work [42,43]. Such analyses would provide crucial insight into whether EP status reflects genuine scholarly breadth and rigor or raises concerns about publication practices. Expanding analyses to include a broader range of journals—particularly emerging and subspecialty titles—would provide a more comprehensive understanding of author productivity across the musculoskeletal research landscape [44]. Incorporating additional databases such as PubMed or Google Scholar may also enhance the robustness of findings by capturing a wider array of publications and reducing database-specific biases. Future studies should also integrate demographic variables such as gender, geographic region, and academic rank to explore questions of equity, diversity, and representation within orthopaedic research [25,26]. Network analyses examining co-authorship and institutional collaborations could further elucidate the social and structural factors that contribute to prolific publishing [18,19]. Additionally, qualitative research approaches may offer deeper insight into the motivations, research practices, and institutional pressures that drive extreme productivity. Given concerns regarding potential trade-offs between publication volume and research integrity, future work should also assess markers of research robustness—such as retractions, corrections, and expressions of concern—among hyperprolific authors [45,46]. Finally, extending analyses beyond the current timeframe and examining longer-term citation patterns could provide valuable information on the sustainability and lasting scientific impact of hyperprolific publishing behaviors. Together, these approaches would contribute to an enhanced understanding of research productivity and its implications to the academic community.

## 5. Conclusions

This study provides a comprehensive analysis of publication patterns among highly productive authors in the fields of sports medicine and musculoskeletal health between 2020 and 2024. Despite comprising less than 0.5% of all contributing authors, HA and AHA accounted for a disproportionate share of total publications. While some authors maintained high output for only brief periods, a smaller subset consistently demonstrated sustained productivity throughout the study period.

Our findings suggest that high publication productivity in this field is often associated with senior authorship roles and involvement in large, collaborative research networks. Importantly, these highly productive authors also exhibited high citation impact, as evidenced by elevated h-index values, indicating that their work achieves both volume and scholarly influence.

Geographically, EP authors were predominantly based in Europe, Asia, and North America, with Germany, Japan, China, and the United States identified as leading contributors. These findings highlight the ongoing concentration of research output within high-income regions and underscore the need for efforts to broaden participation in orthopaedic and sports medicine research globally.

## Supporting information

S1 File Coding data. (ZIP)

S2 Table Appendix A and B. (DOCX)
