# Supplementary material for: Prevalence of hyperprolific authors in sports medicine and musculoskeletal health and implications on research attention
Source: PLoS One. 2026 Mar 18;21(3):e0343827. doi: 10.1371/journal.pone.0343827 (PMC12998802; doi:10.1371/journal.pone.0343827)
Supplement: S2 Table — (DOCX) [file pone.0343827.s002.docx]

# **Appendix A**

## **Hyperprolific (HA) and Almost Hyperprolific (AHA) Authors**

**Appendix Table A1.** Publication counts, maximum author output, and active years for HA and AHA authors between 2020 and 2024.

| **Author Pseudonym** | **Author Type** | **Total Publications**  **(2020-2024)** | **Years with Status** | **Max Annual**  **Publications** | **Study Years** |
| --- | --- | --- | --- | --- | --- |
| **HA author 1** | HA | 1174 | 5 | 262 | 2020, 2021, 2022, 2023, 2024 |
| **HA author 2** | HA | 1082 | 5 | 235 | 2020, 2021, 2022, 2023, 2024 |
| **HA author 3** | HA | 1011 | 5 | 246 | 2020, 2021, 2022, 2023, 2024 |
| **HA author 4** | HA | 657 | 5 | 158 | 2020, 2021, 2022, 2023, 2024 |
| **HA author 5** | HA | 613 | 5 | 152 | 2020, 2021, 2022, 2023, 2024 |
| **HA author 6** | HA | 576 | 5 | 149 | 2020, 2021, 2022, 2023, 2024 |
| **HA author 7** | HA | 575 | 5 | 148 | 2020, 2021, 2022, 2023, 2024 |
| **HA author 8** | HA | 568 | 5 | 128 | 2020, 2021, 2022, 2023, 2024 |
| **HA author 9** | HA | 550 | 5 | 123 | 2020, 2021, 2022, 2023, 2024 |
| **HA author 10** | HA | 524 | 5 | 115 | 2020, 2021, 2022, 2023, 2024 |
| **HA author 11** | HA | 517 | 5 | 120 | 2020, 2021, 2022, 2023, 2024 |
| **HA author 12** | HA | 482 | 5 | 118 | 2020, 2021, 2022, 2023, 2024 |
| **HA author 13** | HA | 481 | 3 | 124 | 2021, 2022, 2023 |
| **HA author 14** | HA | 480 | 4 | 125 | 2020, 2021, 2022, 2023 |
| **HA author 15** | HA | 474 | 5 | 120 | 2020, 2021, 2022, 2023, 2024 |
| **HA author 16** | HA | 473 | 3 | 197 | 2020, 2021, 2022 |
| **HA author 17** | HA | 454 | 5 | 104 | 2020, 2021, 2022, 2023, 2024 |
| **HA author 18** | HA | 447 | 4 | 114 | 2020, 2021, 2022, 2023 |
| **HA author 19** | HA | 439 | 4 | 108 | 2020, 2021, 2022, 2023 |
| **HA author 20** | HA | 437 | 5 | 94 | 2020, 2021, 2022, 2023, 2024 |
| **HA author 21** | HA | 437 | 5 | 105 | 2020, 2021, 2022, 2023, 2024 |
| **HA author 22** | HA | 434 | 5 | 97 | 2020, 2021, 2022, 2023, 2024 |
| **HA author 23** | HA | 431 | 2 | 126 | 2021, 2022 |
| **HA author 24** | HA | 430 | 4 | 95 | 2021, 2022, 2023, 2024 |
| **HA author 25** | HA | 425 | 4 | 109 | 2020, 2021, 2023, 2024 |
| **HA author 26** | HA | 423 | 4 | 96 | 2020, 2021, 2022, 2024 |
| **HA author 27** | HA | 421 | 4 | 98 | 2020, 2021, 2022, 2023 |
| **HA author 28** | HA | 413 | 4 | 111 | 2021, 2022, 2023, 2024 |
| **HA author 29** | HA | 412 | 3 | 108 | 2020, 2021, 2022 |
| **HA author 30** | HA | 410 | 4 | 99 | 2020, 2021, 2022, 2023 |
| **HA author 31** | HA | 396 | 3 | 98 | 2021, 2022, 2023 |
| **HA author 32** | HA | 395 | 4 | 91 | 2021, 2022, 2023, 2024 |
| **HA author 33** | HA | 393 | 4 | 96 | 2020, 2021, 2022, 2023 |
| **HA author 34** | HA | 386 | 4 | 92 | 2020, 2021, 2022, 2023 |
| **HA author 35** | HA | 380 | 3 | 102 | 2020, 2021, 2022 |
| **HA author 36** | HA | 374 | 3 | 110 | 2022, 2023, 2024 |
| **HA author 37** | HA | 374 | 3 | 101 | 2021, 2022, 2023 |
| **HA author 38** | HA | 366 | 2 | 96 | 2022, 2023 |
| **HA author 39** | HA | 366 | 2 | 85 | 2022, 2023 |
| **HA author 40** | HA | 364 | 2 | 83 | 2020, 2021 |
| **HA author 41** | HA | 364 | 3 | 93 | 2021, 2022, 2023 |
| **HA author 42** | HA | 360 | 3 | 81 | 2020, 2021, 2022 |
| **HA author 43** | HA | 357 | 3 | 141 | 2020, 2021, 2022 |
| **HA author 44** | HA | 356 | 2 | 87 | 2022, 2024 |
| **HA author 45** | HA | 356 | 3 | 77 | 2021, 2022, 2023 |
| **HA author 46** | HA | 354 | 2 | 89 | 2022, 2024 |
| **HA author 47** | HA | 350 | 2 | 89 | 2021, 2022 |
| **HA author 48** | HA | 345 | 3 | 101 | 2021, 2022, 2023 |
| **HA author 49** | HA | 343 | 1 | 112 | 2022 |
| **HA author 50** | HA | 335 | 2 | 83 | 2021, 2022 |
| **HA author 51** | HA | 332 | 1 | 84 | 2021 |
| **HA author 52** | HA | 331 | 2 | 75 | 2020, 2021 |
| **HA author 53** | HA | 329 | 2 | 78 | 2021, 2023 |
| **HA author 54** | HA | 325 | 2 | 75 | 2022, 2024 |
| **HA author 55** | HA | 324 | 1 | 92 | 2020 |
| **HA author 56** | HA | 322 | 3 | 89 | 2020, 2021, 2022 |
| **HA author 57** | HA | 319 | 1 | 74 | 2022 |
| **HA author 58** | HA | 317 | 1 | 75 | 2023 |
| **HA author 59** | HA | 315 | 2 | 80 | 2021, 2022 |
| **HA author 60** | HA | 313 | 1 | 79 | 2024 |
| **HA author 61** | HA | 312 | 2 | 82 | 2023, 2024 |
| **HA author 62** | HA | 310 | 1 | 75 | 2022 |
| **HA author 63** | HA | 309 | 1 | 85 | 2020 |
| **HA author 64** | HA | 307 | 1 | 78 | 2021 |
| **HA author 65** | HA | 306 | 1 | 78 | 2021 |
| **HA author 66** | HA | 304 | 1 | 81 | 2021 |
| **HA author 67** | HA | 302 | 2 | 84 | 2021, 2022 |
| **HA author 68** | HA | 301 | 1 | 81 | 2022 |
| **HA author 69** | HA | 299 | 2 | 93 | 2023, 2024 |
| **HA author 70** | HA | 299 | 2 | 76 | 2021, 2022 |
| **HA author 71** | HA | 299 | 1 | 72 | 2024 |
| **HA author 72** | HA | 298 | 1 | 72 | 2024 |
| **HA author 73** | HA | 294 | 3 | 104 | 2020, 2021, 2022 |
| **HA author 74** | HA | 293 | 3 | 96 | 2020, 2021, 2022 |
| **HA author 75** | HA | 293 | 1 | 88 | 2021 |
| **HA author 76** | HA | 290 | 1 | 79 | 2023 |
| **HA author 77** | HA | 289 | 3 | 94 | 2022, 2023, 2024 |
| **HA author 78** | HA | 288 | 2 | 127 | 2023, 2024 |
| **HA author 79** | HA | 286 | 2 | 74 | 2021, 2022 |
| **HA author 80** | HA | 285 | 1 | 80 | 2024 |
| **HA author 81** | HA | 283 | 2 | 77 | 2020, 2021 |
| **HA author 82** | HA | 281 | 1 | 72 | 2021 |
| **HA author 83** | HA | 281 | 3 | 83 | 2020, 2021, 2022 |
| **HA author 84** | HA | 277 | 1 | 78 | 2023 |
| **HA author 85** | HA | 269 | 2 | 78 | 2020, 2021 |
| **HA author 86** | HA | 269 | 1 | 78 | 2022 |
| **HA author 87** | HA | 268 | 3 | 79 | 2022, 2023, 2024 |
| **HA author 88** | HA | 265 | 1 | 115 | 2020 |
| **HA author 89** | HA | 263 | 1 | 117 | 2020 |
| **HA author 90** | HA | 262 | 1 | 72 | 2022 |
| **HA author 91** | HA | 260 | 1 | 73 | 2024 |
| **HA author 92** | HA | 260 | 1 | 74 | 2022 |
| **HA author 93** | HA | 259 | 1 | 75 | 2021 |
| **HA author 94** | HA | 257 | 1 | 88 | 2021 |
| **HA author 95** | HA | 255 | 1 | 77 | 2022 |
| **HA author 96** | HA | 254 | 1 | 74 | 2020 |
| **HA author 97** | HA | 250 | 1 | 73 | 2020 |
| **HA author 98** | HA | 249 | 3 | 88 | 2020, 2021, 2022 |
| **HA author 99** | HA | 247 | 2 | 76 | 2020, 2021 |
| **HA author 100** | HA | 246 | 1 | 83 | 2021 |
| **HA author 101** | HA | 241 | 1 | 73 | 2021 |
| **HA author 102** | HA | 239 | 1 | 72 | 2022 |
| **HA author 103** | HA | 238 | 3 | 86 | 2020, 2021, 2022 |
| **HA author 104** | HA | 238 | 1 | 86 | 2021 |
| **HA author 105** | HA | 236 | 1 | 72 | 2021 |
| **HA author 106** | HA | 233 | 1 | 73 | 2022 |
| **HA author 107** | HA | 232 | 1 | 75 | 2020 |
| **HA author 108** | HA | 223 | 1 | 89 | 2021 |
| **HA author 109** | HA | 213 | 2 | 90 | 2023, 2024 |
| **HA author 110** | HA | 213 | 1 | 80 | 2024 |
| **HA author 111** | HA | 209 | 1 | 77 | 2023 |
| **HA author 112** | HA | 208 | 1 | 73 | 2021 |
| **HA author 113** | HA | 204 | 1 | 81 | 2022 |
| **HA author 114** | HA | 201 | 1 | 85 | 2024 |
| **HA author 115** | HA | 200 | 2 | 84 | 2023, 2024 |
| **HA author 116** | HA | 193 | 1 | 73 | 2024 |
| **HA author 117** | HA | 189 | 1 | 80 | 2023 |
| **HA author 118** | HA | 189 | 1 | 74 | 2020 |
| **HA author 119** | HA | 177 | 1 | 76 | 2024 |
| **HA author 120** | HA | 149 | 1 | 74 | 2024 |
| **HA author 121** | HA | 140 | 1 | 84 | 2024 |
| **HA author 122** | HA | 137 | 1 | 86 | 2024 |
| **HA author 123** | HA | 97 | 1 | 77 | 2024 |
| **HA author 124** | HA | 95 | 1 | 76 | 2024 |
| **HA author 125** | HA | 91 | 1 | 89 | 2024 |
| **AHA author 1** | AHA | 329 | 4 | 71 | 2021, 2022, 2023, 2024 |
| **AHA author 2** | AHA | 322 | 4 | 70 | 2021, 2022, 2023, 2024 |
| **AHA author 3** | AHA | 316 | 3 | 71 | 2021, 2022, 2023 |
| **AHA author 4** | AHA | 314 | 3 | 70 | 2022, 2023, 2024 |
| **AHA author 5** | AHA | 309 | 2 | 71 | 2021, 2022 |
| **AHA author 6** | AHA | 299 | 2 | 71 | 2021, 2023 |
| **AHA author 7** | AHA | 298 | 2 | 67 | 2020, 2023 |
| **AHA author 8** | AHA | 294 | 2 | 69 | 2020, 2024 |
| **AHA author 9** | AHA | 290 | 2 | 65 | 2021, 2023 |
| **AHA author 10** | AHA | 289 | 1 | 62 | 2021 |
| **AHA author 11** | AHA | 288 | 2 | 67 | 2020, 2021 |
| **AHA author 12** | AHA | 286 | 3 | 68 | 2022, 2023, 2024 |
| **AHA author 13** | AHA | 286 | 2 | 67 | 2021, 2024 |
| **AHA author 14** | AHA | 285 | 2 | 70 | 2022, 2024 |
| **AHA author 15** | AHA | 281 | 1 | 70 | 2024 |
| **AHA author 16** | AHA | 280 | 2 | 69 | 2021, 2022 |
| **AHA author 17** | AHA | 279 | 1 | 68 | 2022 |
| **AHA author 18** | AHA | 278 | 1 | 69 | 2021 |
| **AHA author 19** | AHA | 276 | 2 | 66 | 2020, 2021 |
| **AHA author 20** | AHA | 275 | 1 | 65 | 2021 |
| **AHA author 21** | AHA | 272 | 2 | 66 | 2021, 2022 |
| **AHA author 22** | AHA | 272 | 1 | 71 | 2022 |
| **AHA author 23** | AHA | 271 | 2 | 64 | 2022, 2023 |
| **AHA author 24** | AHA | 270 | 2 | 67 | 2021, 2024 |
| **AHA author 25** | AHA | 269 | 2 | 63 | 2021, 2022 |
| **AHA author 26** | AHA | 268 | 1 | 64 | 2020 |
| **AHA author 27** | AHA | 266 | 1 | 61 | 2021 |
| **AHA author 28** | AHA | 265 | 4 | 69 | 2020, 2021, 2022, 2023 |
| **AHA author 29** | AHA | 263 | 1 | 61 | 2021 |
| **AHA author 30** | AHA | 263 | 1 | 63 | 2023 |
| **AHA author 31** | AHA | 260 | 1 | 62 | 2024 |
| **AHA author 32** | AHA | 258 | 1 | 63 | 2024 |
| **AHA author 33** | AHA | 256 | 1 | 62 | 2022 |
| **AHA author 34** | AHA | 254 | 1 | 65 | 2021 |
| **AHA author 35** | AHA | 254 | 2 | 65 | 2022, 2024 |
| **AHA author 36** | AHA | 252 | 1 | 65 | 2022 |
| **AHA author 37** | AHA | 251 | 1 | 66 | 2021 |
| **AHA author 38** | AHA | 249 | 1 | 62 | 2024 |
| **AHA author 39** | AHA | 248 | 1 | 63 | 2020 |
| **AHA author 40** | AHA | 247 | 1 | 62 | 2024 |
| **AHA author 41** | AHA | 246 | 1 | 62 | 2022 |
| **AHA author 42** | AHA | 246 | 1 | 63 | 2020 |
| **AHA author 43** | AHA | 244 | 1 | 64 | 2021 |
| **AHA author 44** | AHA | 244 | 1 | 69 | 2022 |
| **AHA author 45** | AHA | 242 | 1 | 63 | 2021 |
| **AHA author 46** | AHA | 240 | 1 | 66 | 2021 |
| **AHA author 47** | AHA | 238 | 1 | 61 | 2022 |
| **AHA author 48** | AHA | 236 | 1 | 63 | 2021 |
| **AHA author 49** | AHA | 234 | 1 | 62 | 2022 |
| **AHA author 50** | AHA | 232 | 1 | 62 | 2021 |
| **AHA author 51** | AHA | 231 | 1 | 62 | 2022 |
| **AHA author 52** | AHA | 226 | 1 | 67 | 2021 |
| **AHA author 53** | AHA | 225 | 1 | 62 | 2024 |
| **AHA author 54** | AHA | 225 | 2 | 62 | 2022, 2024 |
| **AHA author 55** | AHA | 224 | 1 | 68 | 2022 |
| **AHA author 56** | AHA | 223 | 1 | 71 | 2024 |
| **AHA author 57** | AHA | 221 | 1 | 62 | 2020 |
| **AHA author 58** | AHA | 221 | 1 | 61 | 2021 |
| **AHA author 59** | AHA | 221 | 1 | 61 | 2020 |
| **AHA author 60** | AHA | 220 | 1 | 65 | 2020 |
| **AHA author 61** | AHA | 220 | 2 | 70 | 2022, 2023 |
| **AHA author 62** | AHA | 220 | 1 | 70 | 2021 |
| **AHA author 63** | AHA | 219 | 2 | 70 | 2021, 2022 |
| **AHA author 64** | AHA | 218 | 1 | 63 | 2021 |
| **AHA author 65** | AHA | 213 | 1 | 71 | 2022 |
| **AHA author 66** | AHA | 213 | 1 | 62 | 2022 |
| **AHA author 67** | AHA | 210 | 1 | 71 | 2022 |
| **AHA author 68** | AHA | 210 | 1 | 66 | 2022 |
| **AHA author 69** | AHA | 209 | 2 | 64 | 2020, 2021 |
| **AHA author 70** | AHA | 203 | 1 | 61 | 2022 |
| **AHA author 71** | AHA | 203 | 1 | 61 | 2021 |
| **AHA author 72** | AHA | 202 | 1 | 61 | 2024 |
| **AHA author 73** | AHA | 199 | 1 | 69 | 2024 |
| **AHA author 74** | AHA | 197 | 1 | 62 | 2024 |
| **AHA author 75** | AHA | 197 | 1 | 67 | 2020 |
| **AHA author 76** | AHA | 197 | 1 | 63 | 2021 |
| **AHA author 77** | AHA | 194 | 1 | 64 | 2021 |
| **AHA author 78** | AHA | 193 | 1 | 66 | 2020 |
| **AHA author 79** | AHA | 193 | 1 | 62 | 2021 |
| **AHA author 80** | AHA | 192 | 1 | 64 | 2021 |
| **AHA author 81** | AHA | 191 | 2 | 67 | 2020, 2021 |
| **AHA author 82** | AHA | 188 | 1 | 65 | 2021 |
| **AHA author 83** | AHA | 185 | 1 | 63 | 2023 |
| **AHA author 84** | AHA | 180 | 1 | 66 | 2021 |
| **AHA author 85** | AHA | 180 | 1 | 61 | 2021 |
| **AHA author 86** | AHA | 177 | 1 | 63 | 2024 |
| **AHA author 87** | AHA | 176 | 1 | 61 | 2021 |
| **AHA author 88** | AHA | 176 | 1 | 61 | 2024 |
| **AHA author 89** | AHA | 170 | 1 | 67 | 2024 |
| **AHA author 90** | AHA | 168 | 1 | 67 | 2021 |
| **AHA author 91** | AHA | 166 | 1 | 65 | 2023 |
| **AHA author 92** | AHA | 165 | 1 | 71 | 2024 |
| **AHA author 93** | AHA | 151 | 1 | 67 | 2024 |
| **AHA author 94** | AHA | 148 | 1 | 71 | 2023 |
| **AHA author 95** | AHA | 143 | 1 | 64 | 2020 |
| **AHA author 96** | AHA | 129 | 1 | 65 | 2024 |
| **AHA author 97** | AHA | 111 | 1 | 65 | 2024 |

**Note.** *HA (Hyperprolific Authors)* = Authors publishing ≥72 papers per year (≥1 paper every 5 days); *AHA (Almost Hyperprolific Authors)* = Authors publishing 61-71 papers per year (1 paper every 6 days). Highlighted rows indicate HA authors. Years shown represent the study years (2020-2024) during which the author maintained their respective status.

# **Appendix B**

## **Included Journals**

**Appendix Table B1.** Top 20 CiteScore journals in sports medicine and musculoskeletal health included in the study.

| **Journal Title** | **Scopus Journal ID** |
| --- | --- |
| Nature Reviews Rheumatology | 19700182735 |
| Bone Research | 21100367645 |
| Journal of Bone and Mineral Research | 16214 |
| Osteoarthritis and Cartilage | 26139 |
| Arthritis & Rheumatism | 16450 |
| Annals of the Rheumatic Diseases | 16341 |
| Nature Reviews Endocrinology | 19700182734 |
| Bone & Joint Journal | 21100366539 |
| Journal of Orthopaedic Research | 20982 |
| Arthritis Research & Therapy | 4444309 |
| Clinical Orthopaedics and Related Research | 16808 |
| Spine | 23180 |
| American Journal of Sports Medicine | 16338 |
| Journal of Bone and Joint Surgery (American) | 20948 |
| Knee Surgery, Sports Traumatology, Arthroscopy | 19409 |
| Arthroscopy | 16692 |
| European Spine Journal | 9394 |
| Journal of Shoulder and Elbow Surgery | 21879 |
| Rheumatology | 19882 |
| Seminars in Arthritis and Rheumatism | 15069 |
